# Improving the Performance of Time-Relative GNSS Precise Positioning in Remote Areas

**DOI:** 10.3390/s21010292

**Published:** 2021-01-04

**Authors:** Kaifei He, Duojie Weng, Shengyue Ji, Zhenjie Wang, Wu Chen, Yangwei Lu, Zhixi Nie

**Affiliations:** 1 College of Oceanography and Space Informatics, China University of Petroleum (East China), Qingdao 266580, China; kfhe@upc.edu.cn (K.H.); 19990045@upc.edu.cn (S.J.); sdwzj@upc.edu.cn (Z.W.); niezhixi@upc.edu.cn (Z.N.); 2 Shenzhen Research Institute, The Hong Kong Polytechnic University, Shenzhen 518063, China; 3 Department of Land Surveying and Geo-Informatics, Hong Kong Polytechnic University, Hung Hom, Hong Kong 999077, China; wu.chen@polyu.edu.hk; 4 National Time Service Center Chinese Academy of Sciences, Xi’an 710699, China; luyangwei@ntsc.ac.cn

**Keywords:** time-relative positioning, remote areas, single receiver, broadcast ephemeris

## Abstract

Global navigation satellite systems (GNSS) can attain centimeter level positioning accuracy, which is conventionally provided by real-time precise point positioning (PPP) and real-time kinematic (RTK) techniques. Corrections from the data center or the reference stations are required in these techniques to reduce various GNSS errors. The time-relative positioning approach differs from the traditional PPP and RTK in the sense that it does not require external real-time corrections. It computes the differences in positions of a single receiver at different epochs using phase observations. As the code observations are not used in this approach, its performance is not affected by the noise and multipath of code observations. High reliability is another advantage of time-relative precise positioning because the ambiguity resolution is not needed in this approach. Since the data link is not required in the method, this approach has been widely used in remote areas where wireless data link is not available. The main limitation of time-relative positioning is that its accuracy degrades over time between epochs because of the temporal variation of various errors. The application of the approach is usually limited to be within a time interval of less than 20 min. The purpose of this study was to increase the time interval of time-relative positioning and to extend the use of this method to applications with a longer time requirement, especially in remote areas without wireless communication. In this paper, the main error sources of the time-relative method are first analyzed in detail, and then the approach to improve the accumulated time relative positioning method is proposed. The performance of the proposed method is assessed using both static and dynamic observations with a duration as long as several hours. The experiments presented in this paper show that, among the four scenarios tested (i.e., GPS, GPS/Galileo, GPS/Galileo/BeiDou, and GPS/Galileo/BeiDou/GLONASS), GPS/Galileo/BeiDou performed best and GPS/Galileo/BeiDou/GLONASS performed worst. The maximum positioning errors were mostly within 0.5 m in the horizontal direction, even after three hours with GPS/Galileo/BeiDou. It is expected that the method could be used for positioning and navigation for as long as several hours with decimeter level horizontal accuracy in remote areas without wireless communication.

## 1. Introduction

**Figure 1:**
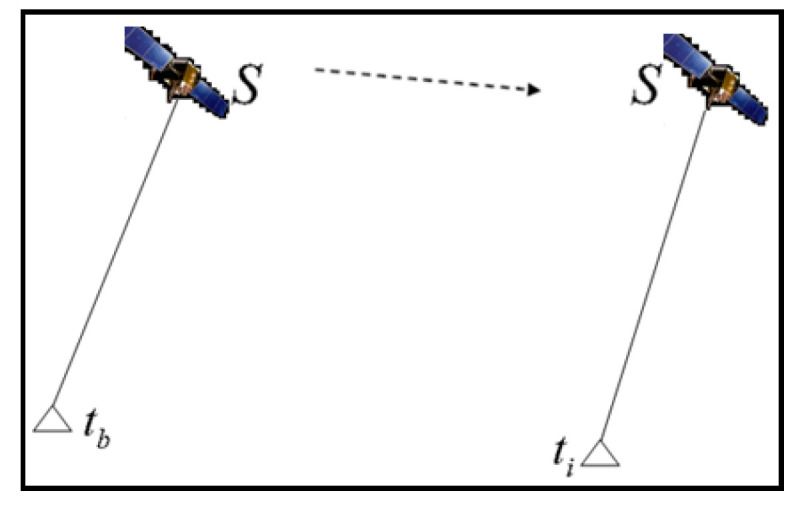
Principle of time-relative method.

Global navigation satellite system (GNSS) precise positions can be determined conventionally using real-time precise point positioning (PPP) and real-time kinematic (RTK) techniques [1,2,3,4,5,6]. These techniques need external support from a data center or reference stations to reduce various GNSS errors. Unlike these techniques, time-relative positioning computes the difference in positions of a single receiver at two epochs, as shown in Figure 1 [7,8,9,10].

Suppose that a GNSS receiver is first placed at station p to collect GNSS data at epoch $t_{b}$ and then the receiver is moved to station q to record the GNSS observations at epoch $t_{i}$. The following two observation equations differenced between satellites can be formed with two sets of observations [11]:

(1) $$A_{p}X_{p}+BN+M_{p}Z=L_{p}$$

(2) $$A_{q}X_{q}+BN+M_{q}Z=L_{q}$$

where $X_{p}$ and $X_{q}$ are the coordinate vectors of stations p and q, respectively; N and Z are the ambiguity vector and zenith tropospheric delay, respectively; $A_{p}$, $A_{q}$, B, $M_{p}$, and $M_{q}$ are their corresponding coefficients; and $L_{p}$ and $L_{q}$ are ionosphere-free phase design vectors for stations p and q, respectively.

When neglecting the differences between $A_{p}$ and $A_{q}$ and the differences between $M_{p}$ and $M_{q}$, the relative position between stations p and q can be calculated by performing the following time-differencing between the two sessions of GNSS data:

(3) $$A_{q}\Delta X_{pq}=\Delta L_{pq}$$

where $\Delta X_{pq}=X_{q}-X_{p}$, and $\Delta L_{pq}=L_{q}-L_{p}$. The absolute position of station q can be determined from the position difference $\Delta X_{pq}$ with respect to station p.

**Figure 2:**
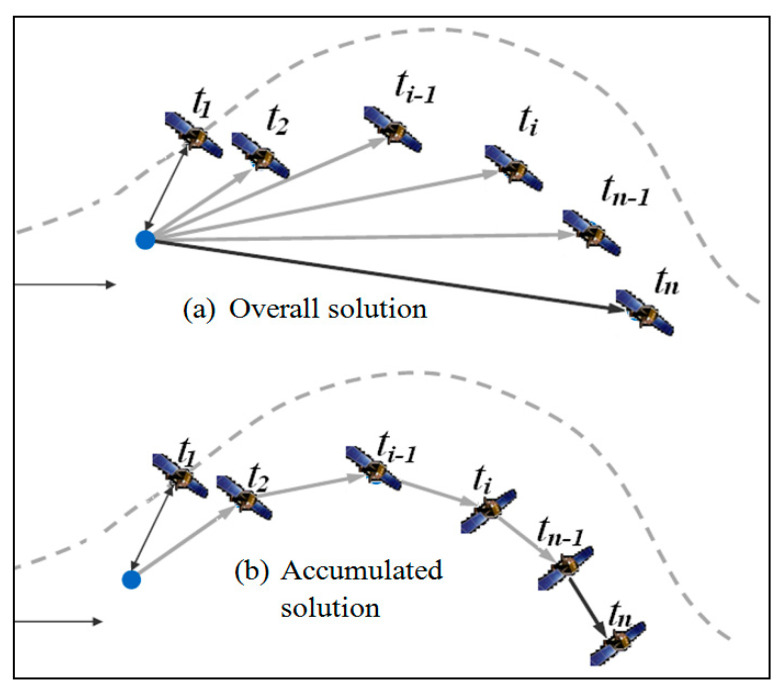
Overall time-relative method vs. accumulated time-relative positioning method.

The method in Figure 1 considers only two sessions of observations, and is often called overall time-relative positioning. Another time-relative positioning method—the accumulated time-relative method—has since been proposed [9]. A comparison of the two methods is presented in Figure 2, and it can be seen that the overall time-relative positioning method takes account of only two sessions of observations, while the accumulated time-relative positioning method considers all epochs during the movement.

Similar to Equations (1) and (2), the following equations (differenced between satellites) can be formed using the accumulated method:

(4) $$A_{1}X_{1}+BN+M_{1}Z=L_{1}\;A_{2}X_{2}+BN+M_{2}Z=L_{2}\;\cdots \cdots \cdots \;A_{i-1}X_{i-1}+BN+M_{i-1}Z=L_{i-1}\;A_{i}X_{i}+BN+M_{i}Z=L_{i}\;\cdots \cdots \cdots \;A_{n}X_{n}+BN+M_{n}Z=L_{n}$$

where, 1, 2, i−1, i and n are epoch indexes.

Neglecting the difference between $A_{i-1}$ and $A_{i}$ and $M_{i-1}$ and $M_{i}$ and performing time-difference between equations of any two adjacent epochs, we obtain

(5) $$A_{2}\Delta X_{1,2}=\Delta L_{1,2}\;A_{3}\Delta X_{2,3}=\Delta L_{2,3}\;\cdots \cdots \cdots \;A_{i}\Delta X_{i-1,i}=\Delta L_{i-1,i}\;\cdots \cdots \cdots \;A_{n}\Delta X_{n-1,n}=\Delta L_{n-1,n}$$

The difference between positions can be determined using the weighted least-squares adjustment method:

(6) $$\Delta X_{1,2}={\left(A_{2}^{T}P_{2}A_{2}\right)}^{-1}A_{2}^{T}P_{2}\Delta L_{1,2}\;\Delta X_{2,3}={\left(A_{2}^{T}P_{2}A_{2}\right)}^{-1}A_{2}^{T}P_{2}\Delta L_{2,3}\;\cdots \cdots \cdots \;\Delta X_{i-1,i}={\left(A_{i}^{T}P_{i}A_{i}\right)}^{-1}A_{i}^{T}P_{i}\Delta L_{i,i}\;\cdots \cdots \cdots \;\Delta X_{n-1,n}={\left(A_{n}^{T}P_{n}A_{n}\right)}^{-1}A_{n}^{T}P_{n}\Delta L_{n-1,n}$$

where $P_{i}$ is the weight matrix based on elevation angle, $P_{i}=Q_{i}^{-1}$.

$$Q_{i}=2\delta_{0}^{2}\left[\begin{array}{ccccc} -1 & 1 & 0 & \ldots & 0\\ -1 & 0 & 1 & \ldots & 0\\ \ldots & \ldots & \ldots & \ldots & 0\\ -1 & 0 & 0 & 0 & 1 \end{array}\right]\left[\begin{array}{cccc} sin^{2}\left(\theta_{1}\right) & 0 & \ldots & 0\\ 0 & sin^{2}\left(\theta_{2}\right) & \ldots & 0\\ \ldots & \ldots & \ldots & \ldots \\ 0 & 0 & \ldots & sin^{2}\left(\theta_{n}\right) \end{array}\right]{\left[\begin{array}{ccccc} -1 & 1 & 0 & \ldots & 0\\ -1 & 0 & 1 & \ldots & 0\\ \ldots & \ldots & \ldots & \ldots & 0\\ -1 & 0 & 0 & 0 & 1 \end{array}\right]}^{T}$$

where $\delta_{0}$ is the standard deviation of carrier phase observation noise, which is generally 3 mm; $\theta_{i}$ is the elevation angle of satellite i.

The precise relative position between epochs 1 and n is given as

(7) $$X_{n}=X_{1}+\Delta X_{1,2}+\Delta X_{2,3}+\ldots +\Delta X_{i-1,i}+\ldots +\Delta X_{n-1,n}$$

Previous studies show that the accumulated time-relative method can provide better positioning accuracy than the overall time-relative method [12] especially under poor data quality; thus, its use is often preferred in practical applications [13,14].

Compared with conventional real-time PPP and RTK, time-relative positioning has many advantages. First, as only phase observations of one receiver are used, the positioning performance is not affected by the noise and multipath from the code observations. Second, the relative position can be acquired immediately after GNSS observations are recorded. Third, the method is reliable, since the ambiguity resolution is not required. Last, it does not require RTK data transmission, so it is a precise positioning method suitable for remote areas without wireless communication. For example, in parts of Tibet or Xinjiang province of China, network RTK or real-time PPP is not available because the GPRS or WiFi data link is not available, yet time-relative positioning can still be widely used when surveying these areas.

Due to the outstanding advantages of the time-relative method, it has been widely used in applications including engineering surveying and mapping [13], ocean navigation [14], airplane navigation [12], altitude determination [7] and seismic monitoring [15,16]. However, the major limitation of time-relative positioning is that its positioning error may be greater than that of the space-relative positioning method. Positioning accuracy degrades rapidly when the duration between observations increases. A time-relative positioning approach with loop misclosure corrections has been proposed to improve its performance [9]. However, this requires the user to return to the starting point after a period of time, and this makes the process of positioning very complex. Therefore, the applications of time-relative positioning are not as popular as RTK and PPP. In practical applications, the time-relative method is mainly used for short time applications, such as one epoch for a cycle slip detection and correction [17], one minute for marine applications [14], several minutes for seismic applications [15], and about twenty minutes for engineering applications [13,16]. Rapid accuracy degradation means the method cannot meet the requirements of precise positioning and navigation applications over a longer timeframe.

In this study, the time-relative positioning method is improved so that it can be used for precise positioning and navigation applications over longer periods of time, especially in remote areas without wireless communication. First, the main error sources of the accumulated time-relative method are analyzed. Then, we propose measures to improve the performance of the positioning method. Finally, the positioning performance of the proposed method is assessed for applications over long time periods and conclusions are drawn.

## 2. Materials and Methods

As the time-relative positioning method is based on a single receiver, the preprocessing process should be the same as PPP in order to remove or reduce as many errors as possible such as carrier phase windup effect, solid earth tide, ocean tide loading, satellite antenna phase center offset, etc. Then, for epochs i−1 and i, the observation equations are given as follows: [18,19]

(8) $$A_{i-1}X_{i-1}+BN+M_{i-1}Z=L_{i-1}+\delta_{orb,i-1}+\delta_{clk,i-1}$$

and

(9) $$A_{i}X_{i}+BN+M_{i}Z=L_{i}+\delta_{orb,i}+\delta_{clk,i}$$

where $\delta_{orb,i}$ is the orbital error and $\delta_{clk,i}$ is the satellite clock error.

Forming a difference between Equations (8) and (9), we can get the following two equations:

(10) $$\left(A_{i}X_{i}-A_{i-1}X_{i-1}\right)+\left(M_{i}-M_{i-1}\right)Z=L_{i}-L_{i-1}+\delta_{orb,i}-\delta_{orb,i-1}+\delta_{clk,i}-\delta_{clk,i-1}$$

and

(11) $$\left(A_{i}X_{i}-A_{i}X_{i-1}+A_{i}X_{i-1}-A_{i-1}X_{i-1}\right)+\Delta M_{i-1,i}Z=\Delta L_{i-1,i}+\Delta \delta_{orb,i-1,i}+\Delta \delta_{clk,i-1,i}$$

After correcting with a tropospheric model, the temporal variation of the remaining tropospheric error is normally very small. Thus, the tropospheric part $\Delta M_{i-1,i}Z$ can be neglected and we will have

(12) $$A_{i}\Delta X_{i-1,i}+\Delta A_{i-1,i}X_{i-1}=\Delta L_{i-1,i}+\Delta \delta_{orb,i-1,i}+\Delta \delta_{clk,i-1,i}$$

Comparing Equation (12) with (3) and (5), we can see that four parts are neglected when forming the time-relative observation equations: $\Delta A_{i-1,i}X_{i-1}$, $\Delta M_{i-1,i}Z$, $\Delta \delta_{orb,i-1,i}$ and $\Delta \delta_{clk,i-1,i}$. The first part relates to the initial coordinate error when linearizing observation equations. The second part is related to tropospheric delay, and the third and fourth parts are related to the orbital and clock errors of broadcast ephemeris, respectively.

As the dry tropospheric delay can be removed almost completely with the tropospheric model and the remaining wet model, after time differencing, generally has a much smaller effect on the positioning than the initial coordinate error and the orbital and clock errors. Therefore, this error can normally be neglected. The effects of the initial coordinate error and the orbital and clock errors will be analyzed in detail in this section.

### 2.1. Initial Coordinate Error

**Figure 3:**
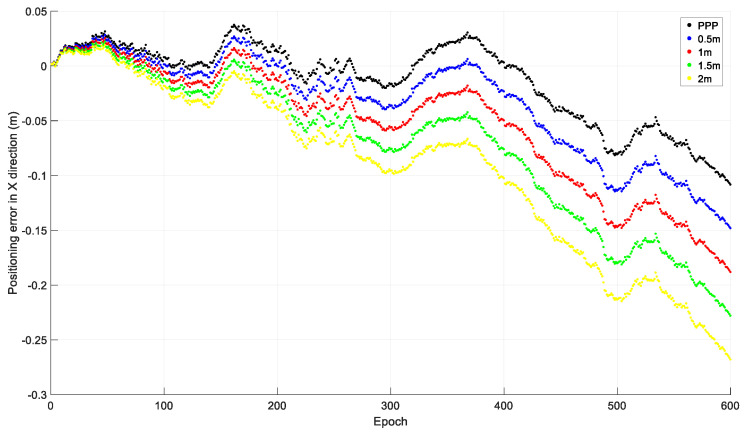
Positioning error in the X direction with different initial coordinates.

**Figure 4:**
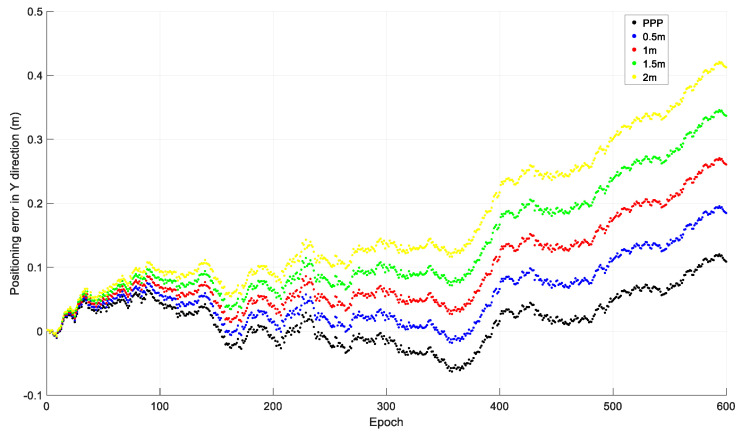
Positioning error in the Y direction with different initial coordinates.

**Figure 5:**
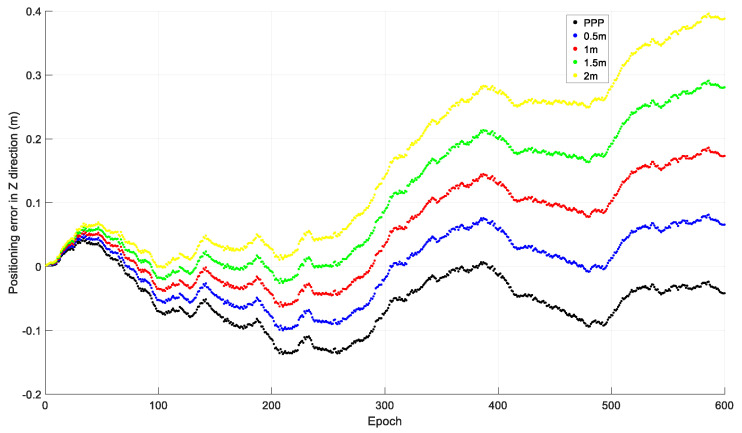
Positioning error in the Z direction with different initial coordinates.

To investigate the effect of the initial coordinate error on time-relative positioning, observations from 20 December 2019 by the Hong Kong CORS station HKLM were downloaded from ftp://ftp.geodetic.gov.hk and the sampling interval was 1 s. The observations of the four GNSSs were collected, and only GPS observations were used in the investigation. The observations were processed with different initial coordinates: The precise coordinates derived from PPP processing; and the PPP coordinates with 0.5 m, 1.0 m, 1.5 m, and 2.0 m added to X, Y, and Z. The relative positions of the other epochs to that of the first epoch were acquired using the accumulated time-relative method. The positioning errors in the X, Y, and Z directions of the first ten minutes, i.e., 600 epochs, are shown in Figure 3, Figure 4 and Figure 5.

From Figure 3, Figure 4 and Figure 5, we can see that the positioning accuracy can be significantly affected by the initial coordinate error. Generally, the larger the initial coordinate error is, the larger the positioning error is. The positioning error also increases quickly with time.

### 2.2. Errors in Broadcast Ephemeris

It is well known that the broadcast ephemeris contains errors. They can be divided into orbit error and satellite clock error. These errors vary with time, and they could not be completely removed through the time differencing operation. The remaining errors had an accumulated effect on the time-relative positioning [5,20]. From Equation (12), we can see that, rather than the size of orbital and clock errors, it is the change of these errors that will affect the time-relative positioning accuracy, and a fast change will make positioning error accumulate rapidly. To reduce their effects on the performance of the time-relative positioning, we can select those satellites with slow changing orbital and clock errors.

We investigated the change rate of orbital and clock errors of broadcast ephemeris of GPS, Galileo, GLONASS, and BeiDou. The investigation was based on the CLK 91 RTS product [21,22] data collected on days 318, 340, 341, 347, and 351–356 in year 2019 (10 days in total and 15 h on average each day). The RTS corrections were processed with the following steps:

- Each type of correction of any satellite was divided into different durations and each duration corresponded to the same IODE or IOD;
- Denoting the maximum and minimum correction values of each duration as Max and Min, the change rate will be R=(Max−Min)/T, where T is the duration length;
- We calculated the average value of R for different durations of each correction of any satellite.

**Figure 6:**
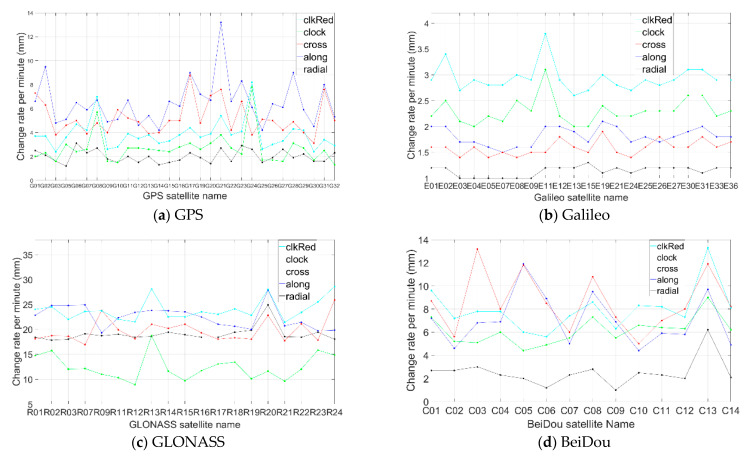
Average change per minute of orbital and clock corrections (**a**: GPS, **b**: Galileo, **c**: GLONASS, **d**: BeiDou).

Figure 6 shows the average change per minute of radial, along, cross, and clock corrections, and also shows the clock correction reduced by the radial one, denoted as “clkRed”. Compared to the along and cross corrections, the radial orbit correction and the clock correction have more effect on positioning. The change rate of the radial correction of Galileo is only slightly higher than 1 mm/min, which is the smallest among the four satellite systems. For GPS, the change rate was around 2 mm/min, while for BeiDou, the change rate of the radical correction was slightly higher, at 2 mm/min. For GLONASS, the change rate was much higher, at almost 20 mm/min.

In terms of the clock correction, the change rates of GPS and Galileo were similar, both slightly over 2 mm/min, and it was around 6 mm/min for BeiDou, while it was more than 10 mm/min for GLONASS. In Figure 6, the “clkRed” curve shows the combined change of radial and clock corrections. The change rate of Galileo was the smallest, at around 3 mm/min; for GPS, it was around 4 mm/min; for BeiDou, it was around 8 mm/min; and for GLONASS, it was more than 20 mm/min.

## 3. Improving the Performance of the Time-Relative Method

Based on the analysis of the main error sources in Section 2, the following measures are proposed to improve the performance of the time-relative positioning:

- Use observations from satellites with slow changing orbital and clock errors. According to Figure 6, Galileo satellites have the slowest changing orbital and clock errors, while the change of orbital and clock errors from GPS satellites is a little faster, and the fastest changing is GLONASS.
- Preprocess observations of any two neighboring epochs with the same parameters of broadcast ephemeris. If preprocessing observations of two neighboring epochs with different parameters of broadcast ephemeris, the corresponding satellite orbital and clock errors can be very different, which would lead to severe time-relative positioning error. When shifting to new broadcast ephemeris information, observations of the current epoch should be preprocessed with the parameters of the old broadcast ephemeris to obtain the relative position between the current epoch and the last epoch. Then, preprocessing should be carried out again with the new broadcast ephemeris to prepare to acquire the relative position of the next epoch.
- Detect sudden changes in satellite position or clock jump with robust estimations. The GNSS satellites can have sudden changes of position, mostly due to satellite maneuvers, especially for BeiDou geostationary satellites. The satellite clock can also jump sometimes. These can be detected based on the residuals of least-squares estimation of Equation (5) or (13); the corresponding observations should be excluded.
- Obtain the precise coordinates of the starting point. The coordinate error of the starting point has a significant effect on the performance of the time-relative method. Therefore, precise coordinates should be acquired before carrying out real-time time-relative positioning. This can be achieved by different techniques such as post-processed precise point positioning (PPP) and long-distance baseline positioning.
- Process observations according to the following procedures:
  ➢ Preprocess the first epoch observations with the precise coordinates of the starting point and obtain the equation $A_{1}{\overset{ˇ}{X}}_{1}+BN+M_{1}Z=L_{1}$.
  ➢ Preprocess observations from the other epochs with the coordinates derived from single point positioning and obtain the equation $A_{i}{\hat{X}}_{i}+BN+M_{i}Z=L_{i}$.
  ➢ Calculate the relative movement, $\Delta {\hat{X}}_{1,2},$ between the first and second epochs based on the equation $A_{2}\Delta X_{1,2}+M_{1,2}Z=\Delta L_{1,2}$.
  ➢ Obtain the precise coordinate of the second epoch by ${\overset{ˇ}{X}}_{2}={\overset{ˇ}{X}}_{1}+\Delta {\hat{X}}_{1,2}$.
  ➢ Reprocess the second epoch observations with the above derived precise coordinates and get the equation $A_{2}{\overset{ˇ}{X}}_{2}+BN+M_{2}Z=L_{2}$.
  ➢ Repeat the above three steps to obtain the precise coordinates of other epochs.

When using for real-time applications, a station with precise coordinates should be established beforehand, then the antenna is setup on the static station and collect observations for a few seconds. After that, move the antenna to any other points to be surveyed while continuously tracking the satellites. Note that, during the process, cycle slip needs to be detected and corrected in real-time.

## 4. Numerical Results

To test the positioning and navigation performance of the proposed method, two tests were carried out. The first test was based on 24 h of static observations from one Hong Kong CORS station, and the second test was based on practical dynamic observations on the near-shore ocean in Qingdao City.

### 4.1. Test Results with Static Observations

**Figure 7:**
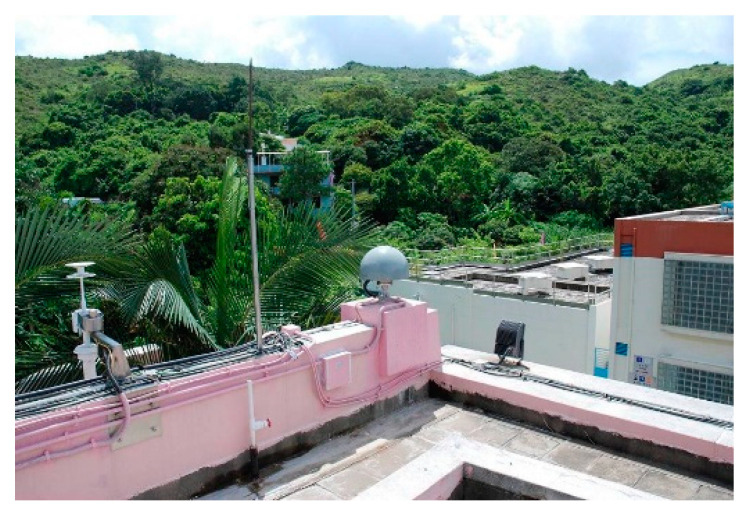
HKLM COSR station.

The 24 h observations the Hong Kong CORS station HKLM collected on 20 December 2019 were processed to assess the accuracy of the proposed time-relative positioning system and to evaluate the time-relative performance for applications over a long period of time simultaneously. Figure 7 shows the environment of the station. The receiver type is Leica GR50 and the sampling interval of the observations is 1 s. The observations for each hour were processed by simulating practical applications.

**Figure 8:**
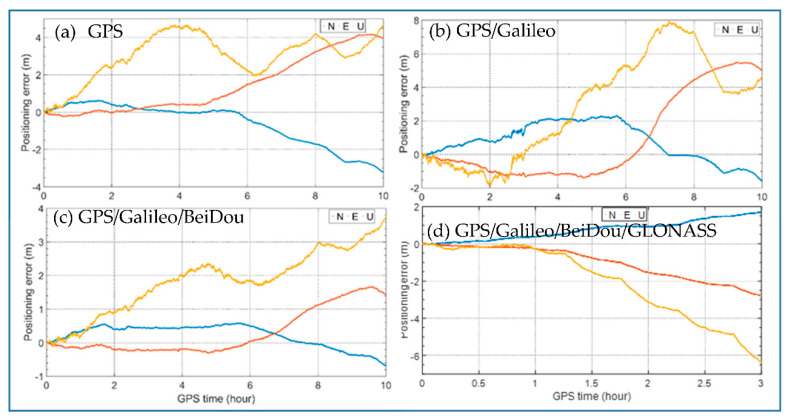
The 10-h time-relative positioning errors with the starting hour 00:00 (**a**: GPS, **b**: GPS/Galileo, **c**: GPS/Galileo/GLONASS, **d**: GPS/Galileo/BeiDou/GLONASS).

We simulated four scenarios with different combinations of satellite systems: GPS alone, GPS/Galileo, GPS/Galileo/BeiDou and GPS/Galileo/BeiDou/GLONASS. The 24 h time-relative positioning errors of these scenarios are calculated with different starting hours. As an example, Figure 8 shows the 10-h positioning errors in north, east and up directions of the four scenarios with starting hour 00:00. North is represented by blue, east by red and up by yellow. We can see that the errors in the vertical direction are the largest which can reach several meters for all these four scenarios. The errors in north and east are much smaller especially for GPS/Galileo/BeiDou, which are below 1 m even after 7 h. Among the four scenarios, GPS/Galileo/BeiDou performs best both in horizontal and vertical directions.

**Figure 9:**
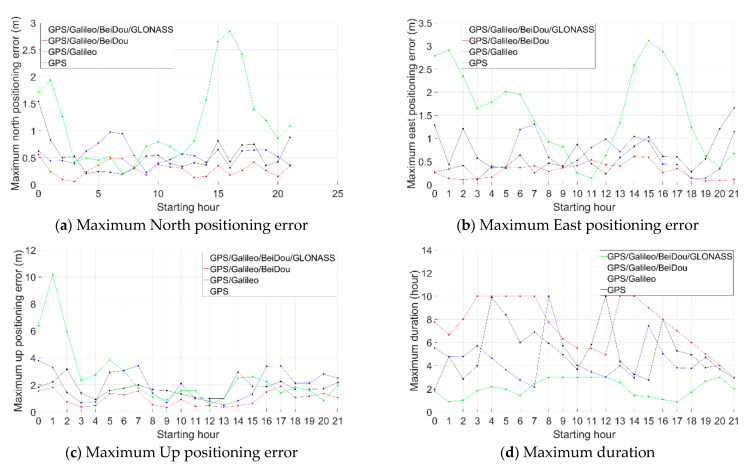
Maximum time-relative positioning error within 3 h (**a**: north, **b**: east, **c**: up) and maximum duration with 1 m precision in both north and east directions (**d**).

For the positioning errors with the other starting hours, see the Appendix A. Figure 9 is the statistics of the maximum time-relative positioning error within 3 h (a, b, c) and the maximum duration within 1 m precision in both north and east directions (d).

As shown in Figure 9, the maximum positioning errors after three hours are mostly less than 0.5 m in horizontal directions when using the proposed method in the scenario of the GPS/Galileo/BeiDou. The best performance was obtained with the GPS/Galileo/BeiDou solution. This is because the performance of the time-relative positioning in terms of accuracy is significantly related to the number satellites used in the solution. The more satellites, the better geometry and the better accuracy obtained.

We can see that for GPS, the positioning performance is not stable, especially in the vertical direction. For example, the positioning error between 02:00–05:00 reached more than 3 m. In the horizontal direction, the positioning errors between 07:00–10:00 and 12:00–15:00 changed slowly and remained within 0.3 m and 0.4 m, while the errors between 00:00–03:00 and 21:00–24:00 reached over 1.5 m. For GPS/Galileo, the positioning performance improved only in the horizontal direction. The reason for this may be that there were only four Galileo satellites observed. For GPS/Galileo/BeiDou, the performance improvement was obvious both in the horizontal and vertical directions. In the horizontal direction, the positioning performance was stable, and positioning errors were all less than 1.0 m, most being less than 0.5 m. In the vertical direction, the positioning errors were generally around 1.0 m. For GPS/Galileo/BeiDou/GLONASS, the performance was generally worse and less stable, especially in the vertical direction, compared to GPS/Galileo/BeiDou. For example, the vertical error between 01:00–04:00 reached almost 10 m and the horizontal errors occasionally reached almost 3 m.

Figure 9d shows the statistics of the longest continuous duration with positioning errors less than 1.0 m both in north and east directions. The GPS/Galileo/BeiDou solution performed best and it lasted generally more than 5 h, occasionally reaching 10 h. The GPS/Galileo/BeiDou/GLONASS solution achieved this low level of error for no more than 3 h.

### 4.2. Practical Dynamic Test Results

**Figure 10:**
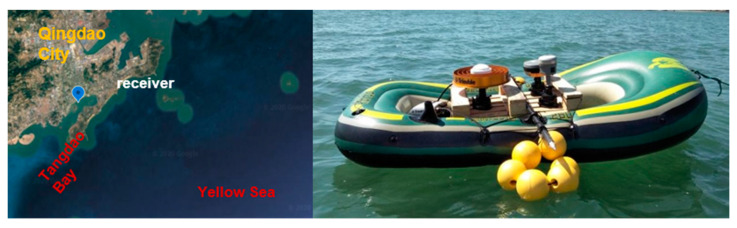
Experimental site (**left**) and setup (**right**).

The performance of the relative positioning method was evaluated in Tangdao Bay, Qingdao City, China on 23 September 2019 (Figure 10). Three GNSS receivers were set up on a boat (Figure 10, right). In this study, only observations from a Trimble ALLOY GNSS receiver with a choke ring antenna were used. In addition, another Trimble ALLOY receiver was set up on the shore with a distance from the shore of no more than 1 km. The observation collection time was from 04:00 to 08:00 (GPS time) and the sampling interval was 1 s.

To obtain the precise position of the Trimble ALLOY receiver on the boat, first, the static observations of the receiver on the shore were processed with the software Bernese 5.2 and the PPP position was obtained with a mm-level RMS. Then, a short baseline was formed between the receiver on the shore and the Trimble ALLOY receiver on the boat, and the precise relative position was acquired with the software RTKLib 2.4.2. Thus, the precise absolute position of the Trimble ALLOY receiver was derived by combing the PPP position of the receiver on the shore and the relative position between these two receivers, which was used to validate the performance of the proposed method in this research.

**Figure 11:**
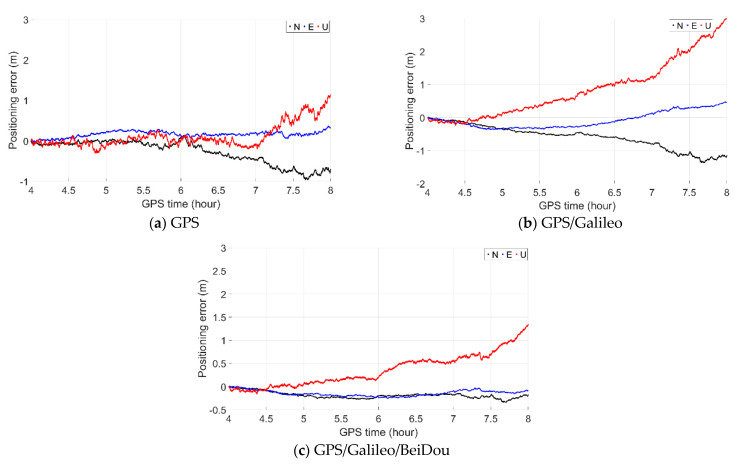
Time-relative positioning errors with (**a**) GPS, (**b**)GPS/Galileo and (**c**) GPS/Galileo/BeiDou.

As shown in Figure 11, the time-relative positioning performance was investigated separately with GPS, GPS/Galileo and GPS/Galileo/BeiDou. Similar to the static test, the positioning performance with GPS/Galileo/BeiDou was the best: All of its errors in the north and east directions were within 0.4 m, even after a duration of as long as 4 h. The vertical errors were no greater than 0.5 m with a duration of 2 h, and generally no greater than 1.0 m with a duration of 4 h.

### 4.3. Analysis and Discussion

**Figure 12:**
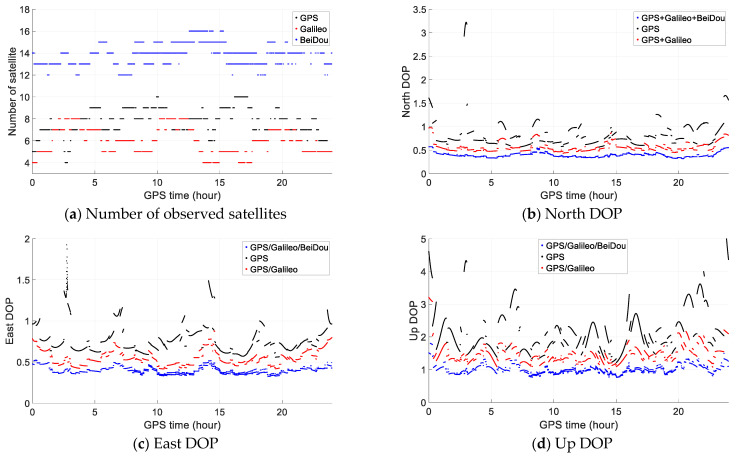
Number of observed satellites (**a**) and north (**b**), east (**c**) and up DOP (**d**).

From the numerical test results, we find that GPS/Galileo/BeiDou performs best. To explore the reason, taking the static test as an example, the number of observed satellites of GPS, Galileo and BeiDou above a cutoff elevation angle of 15° is illustrated in Figure 12 as well as DOP (dilution of precision) in north, east and up directions. We can see that the observed number of GPS satellites is around 7 or 8, it is about 4 for Galileo, while for BeiDou, it is about 14, more than the sum of that of GPS and Galileo. Therefore, the total number of observed satellites of GPS/Galileo/BeiDou is about 25, about double that of GPS/Galileo, which results in good geometry of Dilution of Precision (DOP) especially in east and up directions as shown in Figure 12. With GPS/Galileo/BeiDou, almost all DOP values are below 0.5 in north and east directions and below 1.0 in up direction.

**Figure 13:**
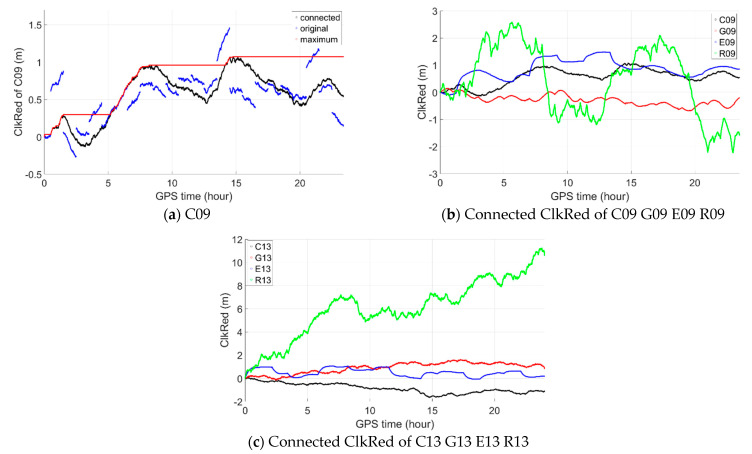
Original, connected and maximum clkRed (**a**: C09, **b**: connected ClkRed of C09 G09 E09 R09, **c**: connected ClkRed of C13 G13 E13 R13).

With the second proposed improving measure by preprocessing observations of any two neighboring epochs with the same parameters of broadcast ephemeris, the orbital and clock errors are in fact connected in the improved time-relative method as shown in Figure 13 (left) by taking BeiDou satellite C09 as an example. The blue one is the original clkRed while the black is the connected. Figure 13 (right) shows the connected clkRed of satellites C13, G13, E13 and R13 as examples and we can see that the size of clkRed may not increase continuously or linearly with time but running up and down generally. So, except for GLONASS, the clkRed error may not reach 2 m or even 1 m after 24 h as illustrated by the red which gives the maximum clkRed error till current epoch.

**Figure 14:**
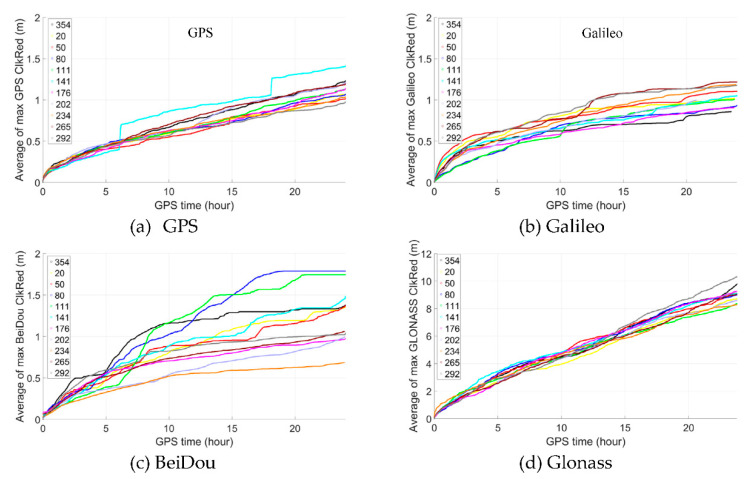
The average of the maximum clkRed of (**a**) GPS, (**b**) Galileo, (**c**) BeiDou and (**d**) GLONASS satellites.

As it is the maximum size of clkRed error that affect the maximum positioning error most with the improved time-relative method and, Figure 14 shows the average value of the maximum clkRed till current epoch of all GPS, Galileo and BeiDou satellites on different days of year, including 354 of year 2019 (the experimental date of above static test), 20, 50, 90, 111, 141, 176, 202, 234, 265 and 292 of year 2020. We can see that, for GPS, it is very stable generally, the average of the maximum clkRed does not exceed 0.5 m within 5 h and only reach about 1.0 m after 20 h. For Galileo, though not as stable as GPS, it reaches about 0.5 m after 5 h and generally no more than 1.0 m after 15 h. For BeiDou, though the most unstable one, but the average of the maximum clkRed does not exceed 0.5 m generally within 5 h and only reach about 1.0 m after 10 h. Therefore, we can expect the repeatability of similar performance on the other dates. While for GLONASS, the average of the maximum clkRed is much bigger than that of the other three GNSS, which should be the reason that GPS/Galileo/BeiDou/GLONASS has poorer performance.

**Figure 15:**
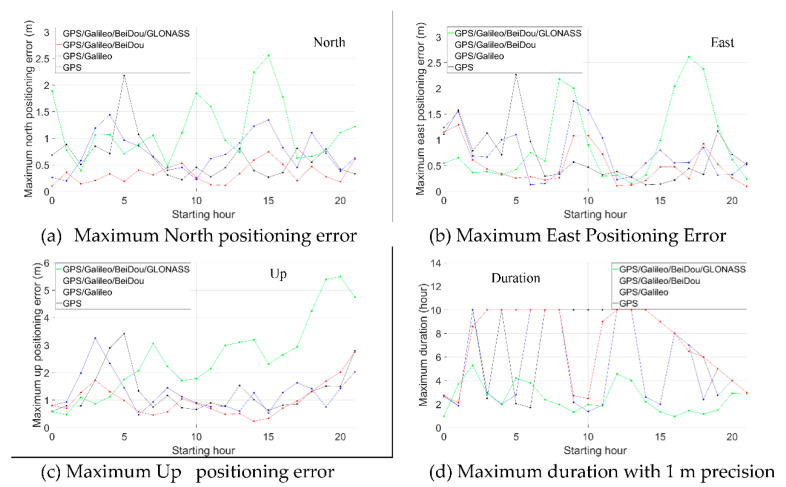
Maximum time-relative positioning error within 3 h (**a**: north, **b**: east, **c**: up) and (**d**) maximum duration with 1 m precision in both north and east directions of station GOPE.

To demonstrate the repeatability of the performance on the other dates, 24-h GNSS observations of IGS station GOPE (49.9° N, 14.8° E) on 21 September 2020 are downloaded and processed same to that of station HKLM. Figure 15 shows the maximum positioning errors within 3 h and the maximum duration with 1 m precision in both north and east directions. We can see that the performance is similar to that of HKLM. Also, GPS/Galileo/BeiDou performs best and GPS/Galileo/BeiDou/GLONASS performs worst. And for GPS/Galileo/BeiDou, most of the maximum positioning errors are below 0.5 m in both north & east directions and most of the maximum duration with 1 m precision in both north & east directions exceeds 6 h.

## 5. Conclusions

The purpose of this study was to provide a real-time precise relative positioning method for surveying and navigation applications in remote areas, such as parts of the Tibet or Xinjiang provinces of China, where GPRS or a WiFi data link is not available. The main error sources were first analyzed in detail and measures to improve the accumulated time relative method were proposed. Then, the positioning performance of the proposed measures was assessed. The numerical results over a long period of time with both static and dynamic observations showed that the time-relative positioning performance was not stable using GPS or GPS/Galileo, and especially with GPS/Galileo/BeiDou/GLONASS. With the multiple constellation of GPS/Galileo/BeiDou, the maximum positioning errors were less than 1.0 m, and most were within 0.5 m in a horizontal direction, even with a duration as long as three hours. In a vertical direction, most errors were around 1.0 m with a duration of up to three hours. We can see that the improved time-relative method can be used for positioning and navigation for as long 5 h with a decimeter level of horizontal accuracy, which can meet the demanding positioning requirements of some applications including engineering surveying and large-scale mapping, for example, 1:500, especially in remote areas.

## Figures and Tables

**Figure A1:**
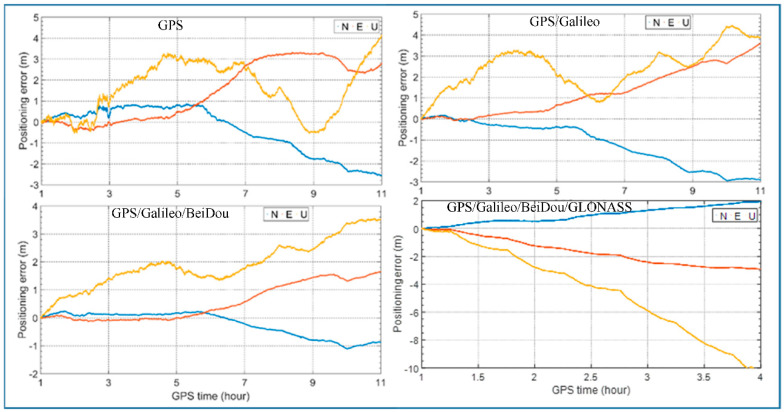

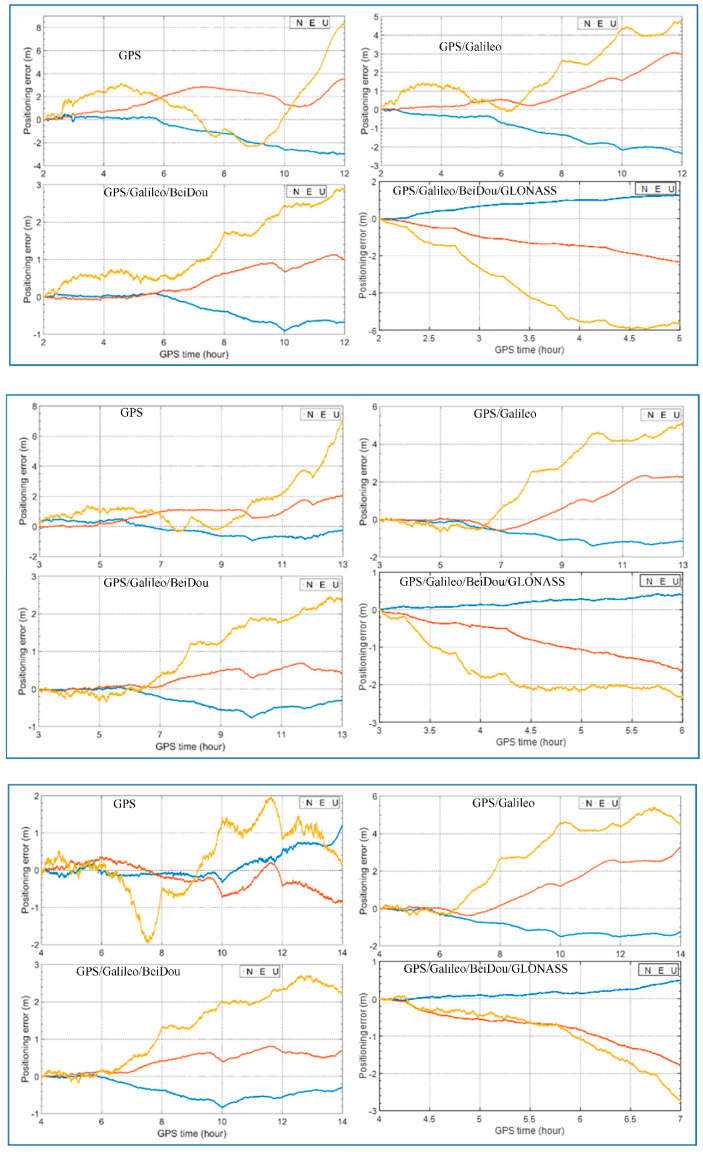

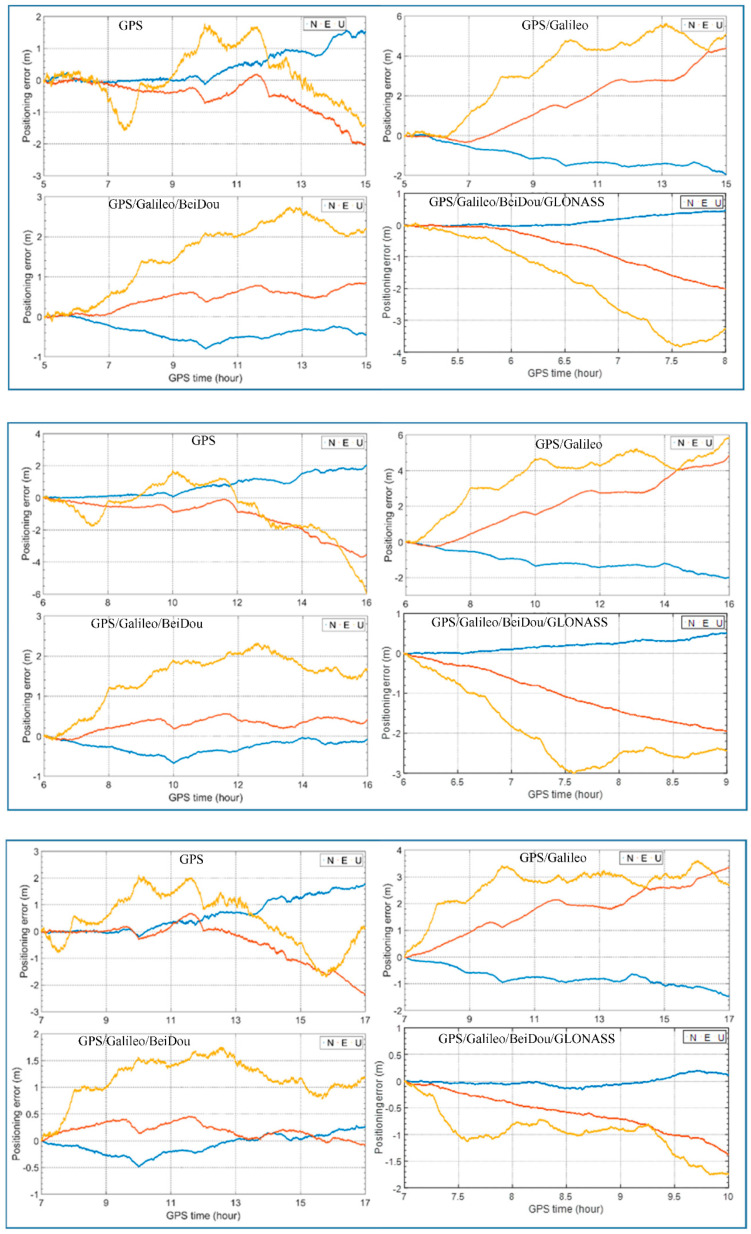

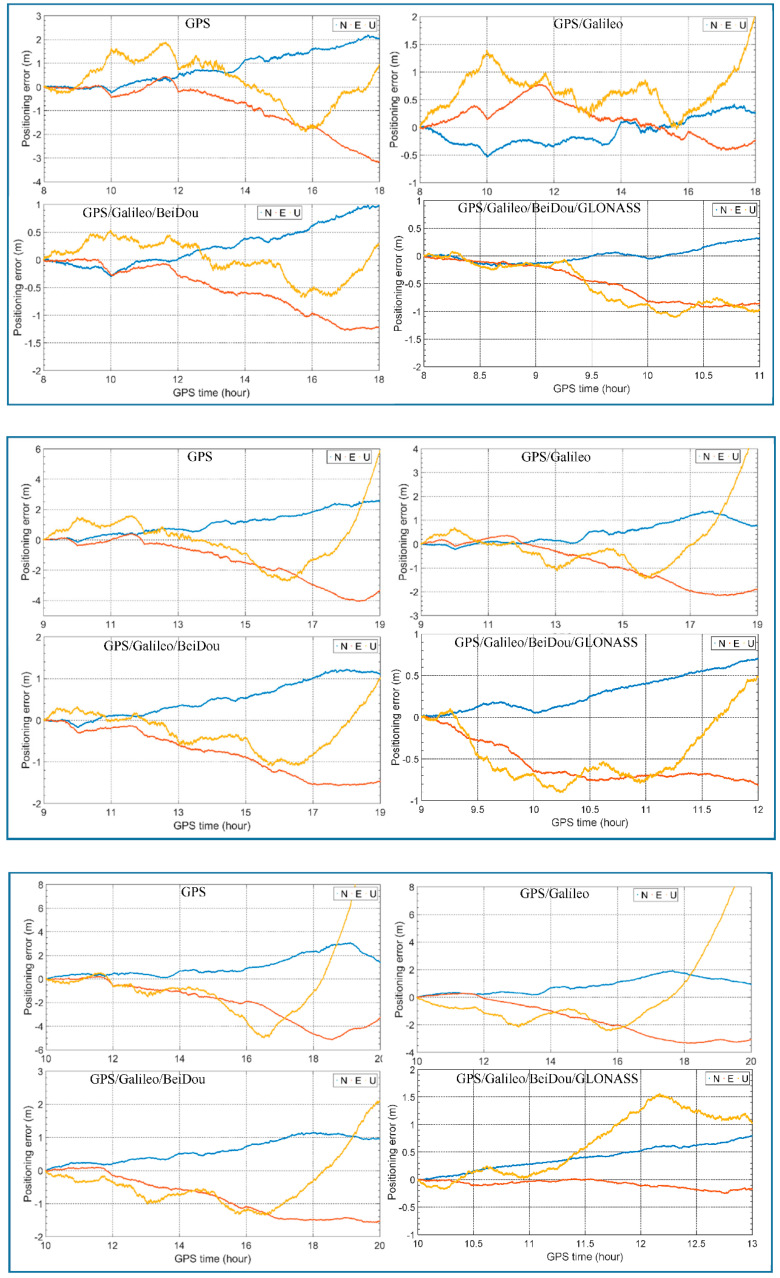

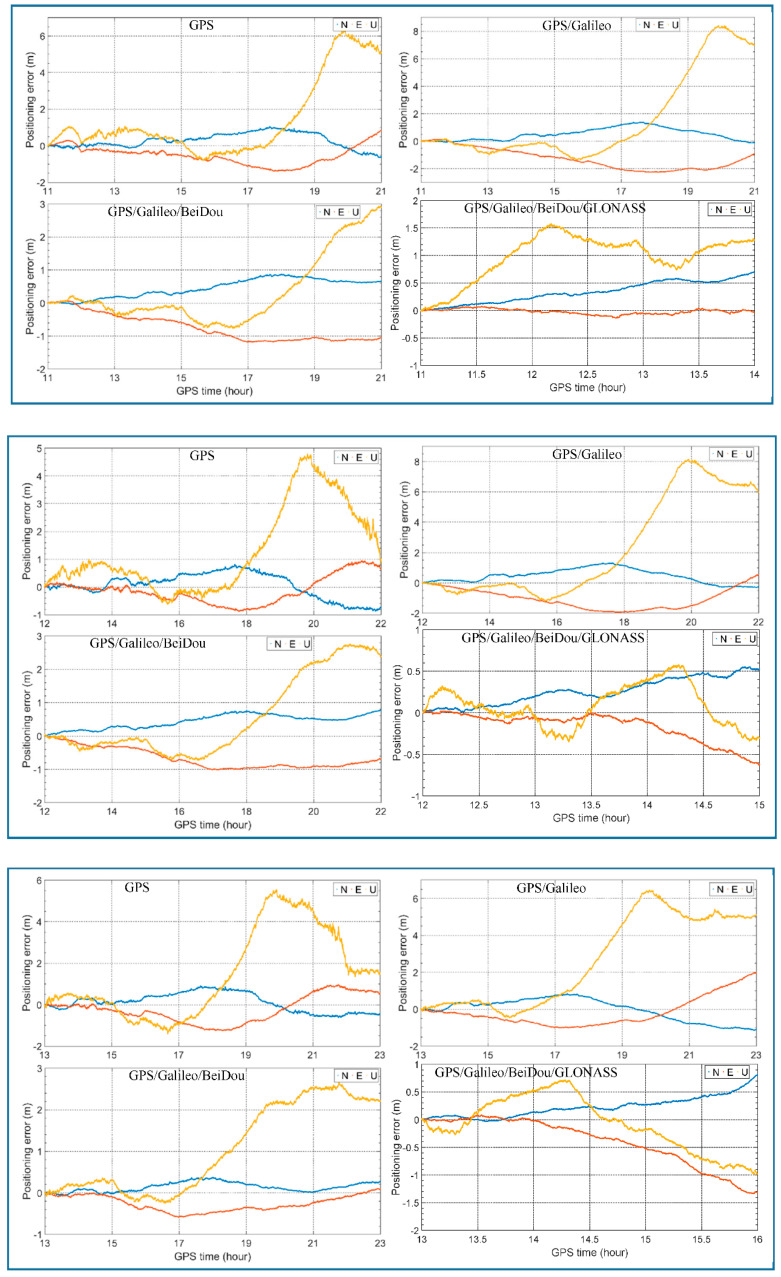

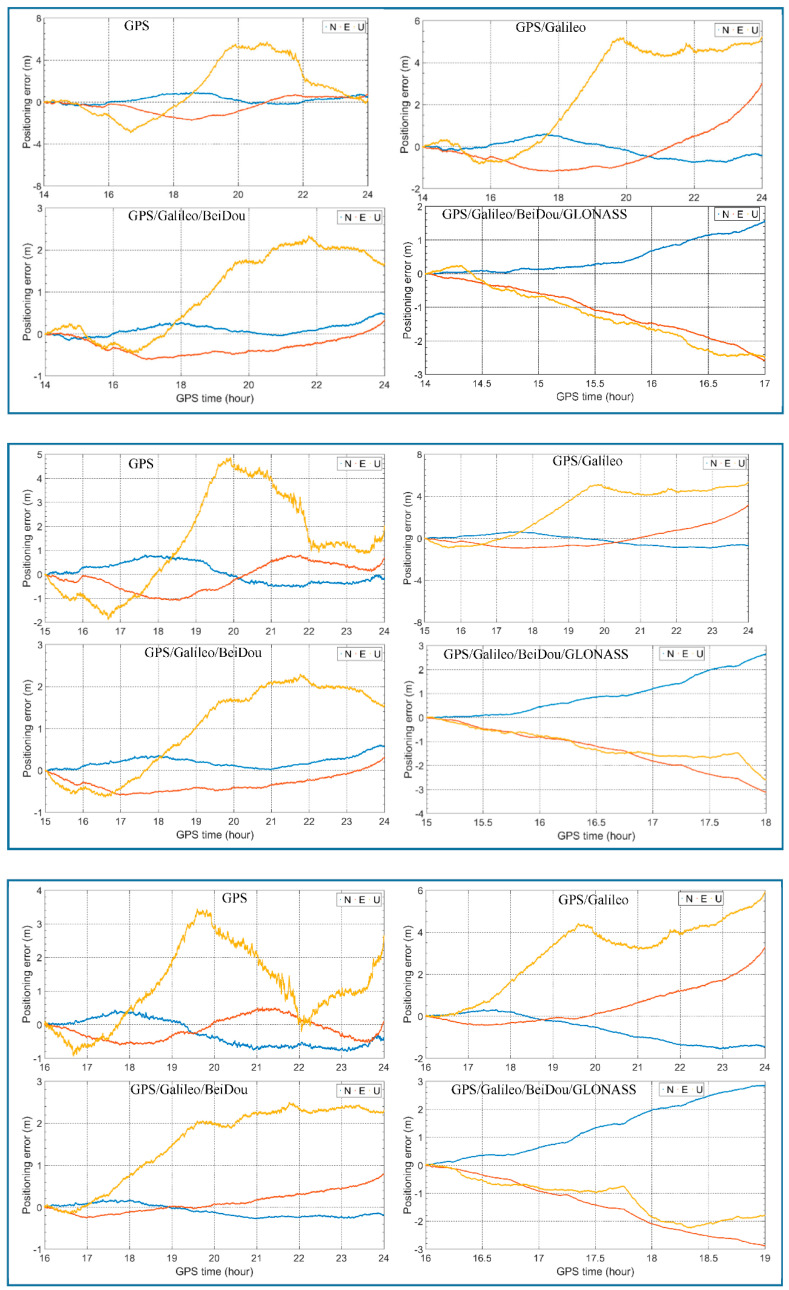

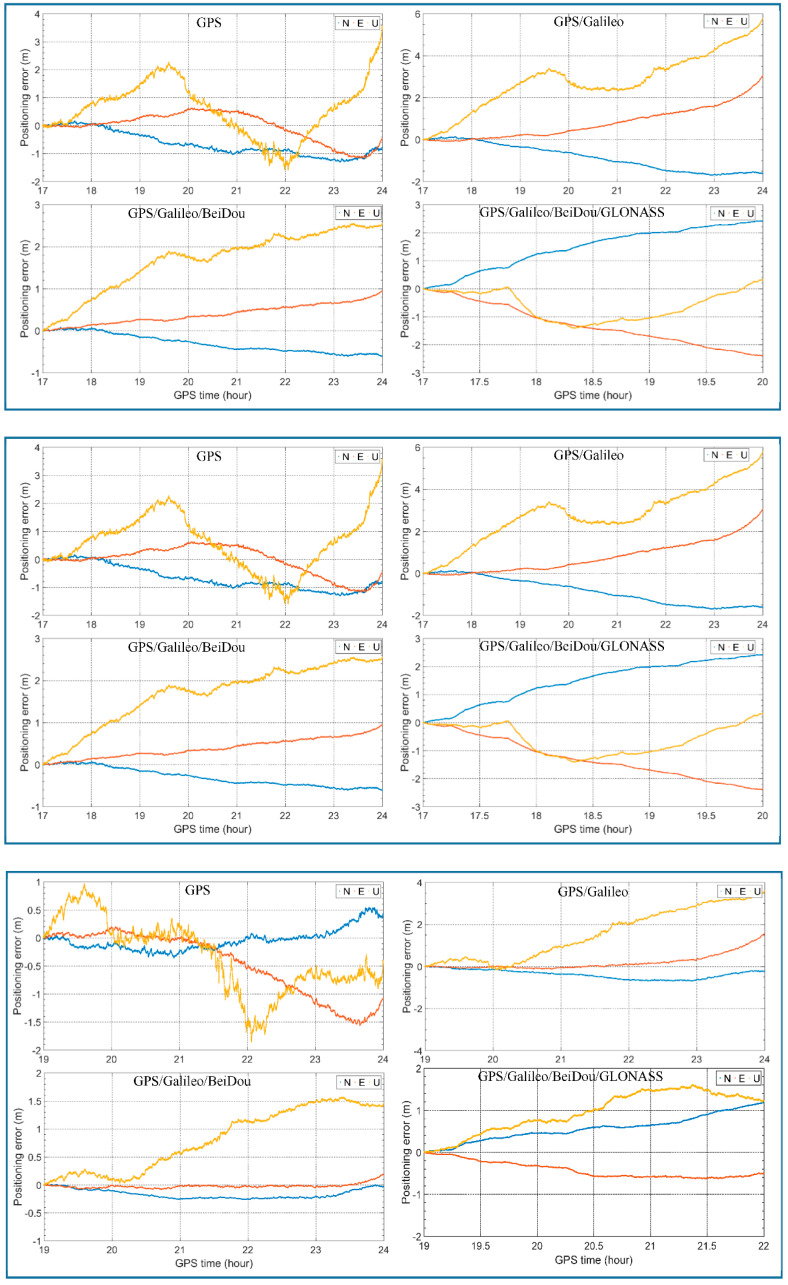

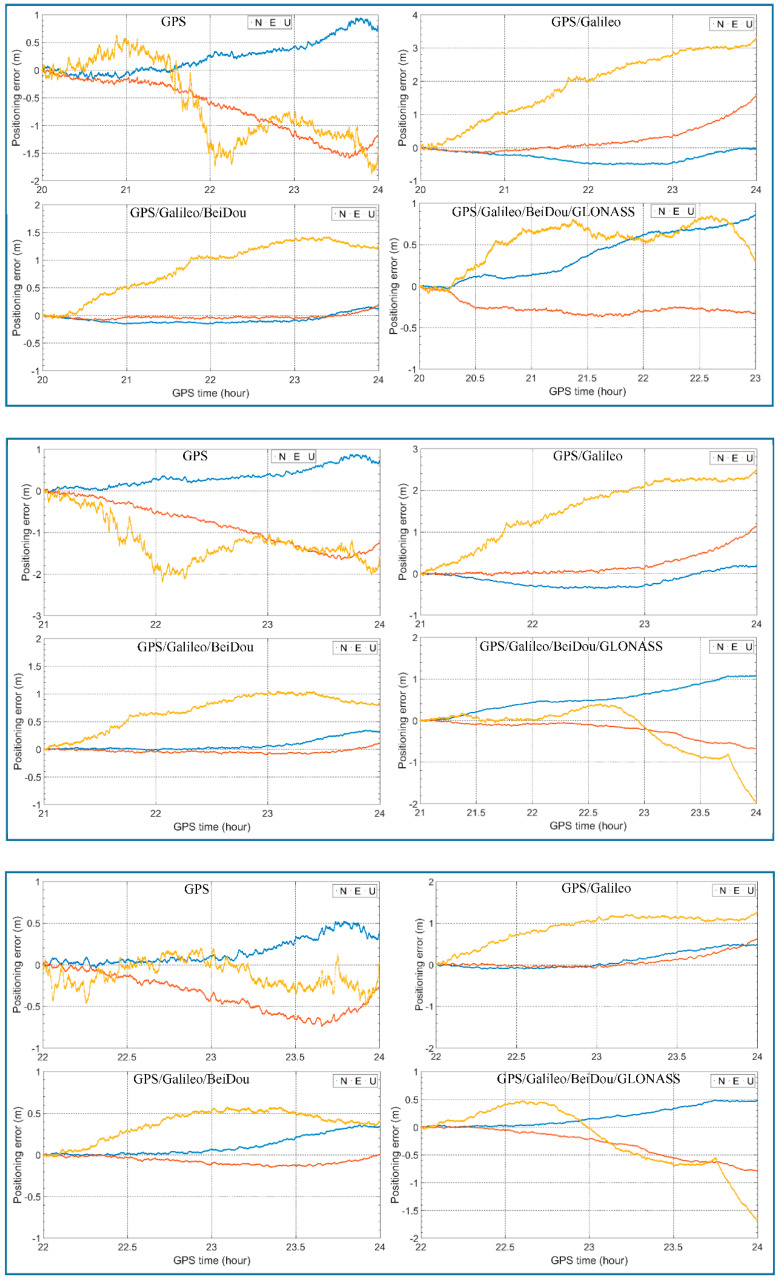

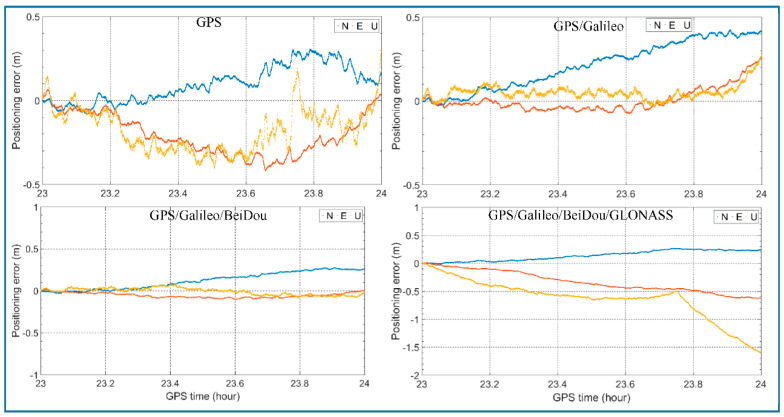
The time-relative positioning errors with different scenarios in 24 h.

## Data Availability

All data included in this study are available upon request by contactwith the corresponding author.
